# Multi-Functional Nano-Doped Hollow Fiber from Microfluidics for Sensors and Micromotors

**DOI:** 10.3390/bios14040186

**Published:** 2024-04-10

**Authors:** Yanpeng Wang, Zhaoyang Wang, Haotian Sun, Tong Lyu, Xing Ma, Jinhong Guo, Ye Tian

**Affiliations:** 1 College of Medicine and Biological Information Engineering, Northeastern University, Shenyang 110169, China; wangyanpeng1001@163.com (Y.W.); wangzhaoyang0908@163.com (Z.W.); s1328607240@163.com (H.S.); verapetrova@163.com (T.L.); 2 School of Materials Science and Engineering, Harbin Institute of Technology (Shenzhen), Shenzhen 518055, China; maxing@hit.edu.cn; 3 School of Sensing Science and Engineering, Shanghai Jiao Tong University, Shanghai 200240, China; 4 Foshan Graduate School of Innovation, Northeastern University, Foshan 528300, China

**Keywords:** hollow fibers, nano-doped hydrogels, microfluidics, wearable sensors, micromotors

## Abstract

Nano-doped hollow fiber is currently receiving extensive attention due to its multifunctionality and booming development. However, the microfluidic fabrication of nano-doped hollow fiber in a simple, smooth, stable, continuous, well-controlled manner without system blockage remains challenging. In this study, we employ a microfluidic method to fabricate nano-doped hollow fiber, which not only makes the preparation process continuous, controllable, and efficient, but also improves the dispersion uniformity of nanoparticles. Hydrogel hollow fiber doped with carbon nanotubes is fabricated and exhibits superior electrical conductivity (15.8 S m^−1^), strong flexibility (342.9%), and versatility as wearable sensors for monitoring human motions and collecting physiological electrical signals. Furthermore, we incorporate iron tetroxide nanoparticles into fibers to create magnetic-driven micromotors, which provide trajectory-controlled motion and the ability to move through narrow channels due to their small size. In addition, manganese dioxide nanoparticles are embedded into the fiber walls to create self-propelled micromotors. When placed in a hydrogen peroxide environment, the micromotors can reach a top speed of 615 μm s^−1^ and navigate hard-to-reach areas. Our nano-doped hollow fiber offers a broad range of applications in wearable electronics and self-propelled machines and creates promising opportunities for sensors and actuators.

## 1. Introduction

Functional hydrogel fiber has attracted widespread attention due to its broad application prospects. With the advancement of technology, functional hydrogel fiber has been used for drug delivery [1,2], cell culture [3,4,5], tissue engineering [6,7], substance separation [8,9,10], wearable electronic devices [11,12,13], and actuators [14,15], showing excellent biocompatibility, adsorption, sensing properties, and actuation ability. However, the underlying hydrogel matrix cannot possess the above capabilities, so a series of nanoparticles have been successfully combined with polymer networks to obtain nano-doped hydrogels [16,17], including carbon nanotubes [18,19,20], graphene [21], metal nanoparticles [22], and transition metal carbide nanosheets [12,23]. It has been shown that the doping of nanomaterials in fiber helps to improve their mechanical properties [24]. Compared to bulk hydrogels, hollow hydrogel fibers have a larger specific surface area, which allows many reactions to proceed more fully. Nano-doped hydrogel functional fibers are receiving more and more attention because the fibers exhibit excellent weave ability, so they can be customized into arbitrary shapes with high softness.

However, conventional preparation methods for obtaining nano-doped hollow fibers have certain limitations. The conventional template method is not able to prepare ultra-long fibers continuously [25,26], and the electrostatic spinning method [27,28] is unable to ensure the nanoparticle agglomeration during the preparation process due to electrostatic force and van der Waals force [29]. The fiber impregnation method [30,31] is unable to ensure that the nanoparticles will not be dislodged during subsequent use. Microfluidics has been developed over decades as a mature technology and is widely used in many industries and basic research areas [32,33,34,35,36]. Since the fluids in co-flow microfluidic systems all flow in a laminar flow at low speed [37], the possibility of nanoparticle collisions is low and the adhesion between particles is weak [38], which ensures that they do not aggregate during the preparation process. However, the microfluidic techniques employed in the existing literature usually rely on an internal curing process to form hollow fiber structures [32,39]. This approach, although feasible to a certain extent, is prone to triggering channel blockage, since the curing reaction takes place inside the system, thus leading to discontinuity in the preparation process. Therefore, there is an urgent demand to use microfluidic methods to fabricate nano-doped hollow fibers in a simple, smooth, stable, continuous, well-controlled manner without system blockage.

In this study, we chose a microfluidic method to prepare nano-doped hollow fiber using coaxial glass capillary chips in a simple, smooth, stable, continuous, and well-controlled manner. Due to the curing strategy outside the microfluidic system, our microfluidic method greatly reduces system blockage. The rapid gelation reaction between sodium alginate and calcium ions ensures that the nanoparticles are firmly anchored inside the fibers, avoiding degradation of fiber properties due to displacement of nanoparticles during use. Therefore, we doped carbon nanotubes in hydrogel hollow fibers and prepared wearable fiber sensors using the hollow fibers. The hollow fibers have a high conductivity of 15.8 S m^−1^, a high stretch rate of 342.9%, and high sensitivity at high deformation rates. Iron tetroxide nanoparticles were incorporated into hydrogels to create magnetic-driven micromotors. These micromotors not only enable trajectory-controlled motion but can also, due to their small size, pass through narrow passages, giving the fibers the ability to have adjustable physical properties with precise motion control. Additionally, to enhance the functional capabilities of these fibers, manganese dioxide (MnO_2_) nanoparticles were intricately embedded within their walls, thereby transforming them into self-propelled micromotors. Upon exposure to a hydrogen peroxide environment, these micromotors demonstrated remarkable autonomous mobility, achieving a maximum velocity of 615 μm s^−1^. This unique attribute not only facilitates their navigation through challenging and confined spaces, but also endows the fibers with heightened environmental responsiveness, autonomous motion capabilities, and an inherent mechanism for energy generation. The results show that hollow fibers containing different functional nano-dopants prepared by microfluidic technology have successfully achieved the multifunctionalities of hollow fibers. This technique not only permits the preparation of multiple functional hollow fibers on the same preparation platform, but, more importantly, it demonstrates the advancement and flexibility of microfluidics in material design and preparation. Especially in wearable electronic devices, soft robotics, and other potential applications, these hollow fibers show promising applications due to their light weight, high elasticity, and customizable chemical and physical properties, and demonstrate practicality and innovation in these fields of application.

## 2. Materials and Methods

### 2.1. Materials

Sodium alginate (NaA, Sigma Industrial Corporation, Phoenix, AZ, USA) from brown algae, polyvinyl alcohol (PVA, Mw: 13,000–23,000, hydrolyzed: 87–89%, Sigma Industrial Corporation, Phoenix, AZ, USA) and calcium chloride (CaCl_2_ anhydrous, powder, AR, 96%, Macklin Industrial Corporation, Shanghai, China), polyethylene glycol (PEG, Mw: 6000, Aladdin Industrial Corporation, Shanghai, China), hydroxypropyl methyl cellulose (HPMC, Shandong Yousuo Chemical Technology Co., Ltd., Jinan, Shandong, China), methyl cellulose (MC, Shandong Yousuo Chemical Technology Co., Ltd., Jinan, Shandong, China), ferric oxide nanoparticle dispersion (Fe_3_O_4_, VK-EF01W, 20% solid content, 20 nm–30 nm, Jinan Zhiding Welding Material Co., Ltd., Jinan, Shandong, China), large diameter carboxylate multi-walled carbon nanotubes (CNTs, JCMW CC4, Shandong Jiacai Technology Co., Ltd., Jinan, Shandong, China), Lithium chloride (LiCl anhydrous, powder, AR, 98%, Aladdin Industrial Corporation, Shanghai, China), manganese dioxide (MnO_2_, powder, GR, ≥90%, Aladdin Industrial Corporation, Shanghai, China), hydrogen peroxide (H_2_O_2_, Bangjian pharmaceutical chain Co., Ltd., Bangjian, China).

### 2.2. Methods

#### 2.2.1. Design of the Microfluidic Chip

A micropipette puller (PUL-100, World Precision Instruments, Inc., Sarasota, FL, USA) was used to prepare the capillary-based microfluidic device. Two cylindrical capillaries (World Precision Instruments, Inc., Sarasota, FL, USA) with inner diameters of 0.58 mm and outer diameters of 1 mm were tapered by the micropipette puller to obtain an injection section and a collection section, respectively. Their tips were polished to the desired diameter using fine sandpaper. The capillary microfluidic device consists of a glass slide and a group of nested glass capillaries. Two injection needles were encapsulated at the capillary junction. Two cylindrical capillaries with tips were placed in a square glass capillary (1.4 mm × 1.1 mm, Beijing Chengteng Equipment Co., Ltd., Beijing, China), aligned coaxially, and glued together with AB glue (5 Minute Epoxy, Deli Group Co., Ltd., Ningbo, Zhejiang, China) to obtain nested capillary groups. At the end of the collection section, a petri dish was placed, filled with calcium chloride (5 wt%) solution to solidify the generated hollow fiber.

#### 2.2.2. Fabrication of Hollow Fiber

**Scheme 1:**
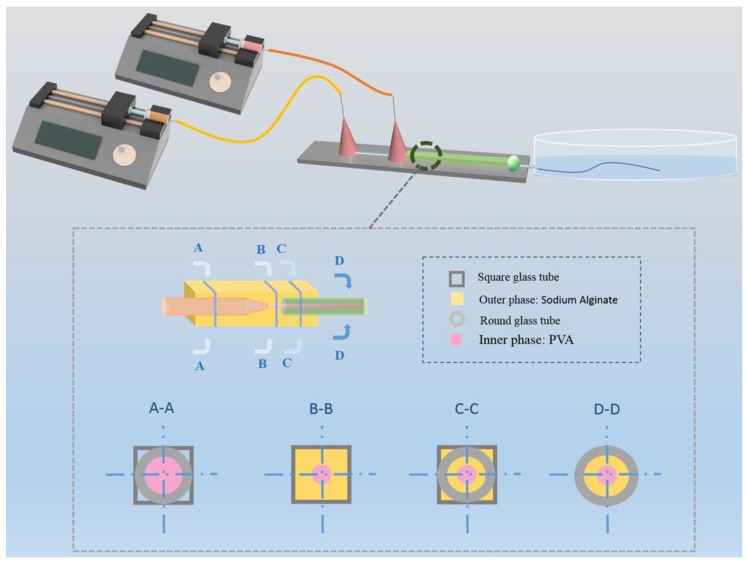
Schematic diagram showing the experimental setup for the fabrication of hollow fiber. A–A, B–B, C–C, and D–D each correspond to the magnified cross-sectional views of specific combinations in different positions of the channel schematic shown above in an academic context.

Sodium alginate (NaA) was dissolved completely into a 2.5 wt% sodium alginate solution with heating and stirring in a constant temperature water bath at 70 °C. Subsequently, a 10 wt% polyvinyl alcohol solution was prepared. For easy observation, we added a little red pigment to the polyvinyl alcohol solution to distinguish the inner and outer phases. Next, the solutions were introduced into the microfluidic device via a syringe pump (Baoding Lange constant flow pump Co., Ltd., BaoDing, HeBei, China). Before fabrication, NaA was injected into the device to expel the air in the device and to improve the stability of the system. During fabrication, the flow rate of the inner phase should be slower than that of the outer phase. The flow rate of the outer phase was adjusted in the range of 0.5 mL h^−1^–1.2 mL h^−1^, and the flow rate of the inner phase was adjusted in the range of 0.1 mL h^−1^–1.0 mL h^−1^. Finally, a 5 wt% CaCl_2_ solution was used as a coagulation bath to solidify the hollow fibers. The prepared fibers were extracted with tweezers and washed in ultrapure water to remove the PVA solution and obtain the hollow fibers. As a control experiment, we also fabricated hollow fibers with PEG (10 wt%), MC (2 wt%), and HPMC (1.6 wt%) as the inner phase, respectively, using the same process (Scheme 1).

Fabrication of HollowFiber-CNTs. In the preparation of CNT-doped hollow fibers, NaA (2.5 wt%), doped with 1 wt%, 2 wt%, 3 wt%, 4 wt%, and 5 wt% CNT dispersion, was used as the outer phase solution, and PVA (10 wt%) solution was used as the inner phase solution to fabricate CNT-doped hollow fiber. The other preparation procedures were the same as above. The CNT-doped hollow fibers were named HollowFiber-CNTs. The generated HollowFiber-CNTs were immersed in 1 wt% lithium chloride solution for 2 h to enhance their conductivity, and then the excess salt solution on the fiber surface was washed with ultrapure water for subsequent preparation.

Fabrication of HollowFiber-Fe_3_O_4_. We dispersed water-based magnetic fluid containing Fe_3_O_4_ nanoparticles into 2 wt% NaA solution (volume fraction 12.5%) as the outer phase [40,41] and PVA (10 wt%) as the inner phase, and the velocity ratio of the inner and outer phases was set at 0.65 mL h^−1^:1.2 mL h^−1^ to prepare hollow fibers with a uniform distribution of magnetic nanoparticles. The other preparation procedures were the same as above. The hollow fibers with a uniform distribution of magnetic nanoparticles were named HollowFiber-Fe_3_O_4_.

Fabrication of HollowFiber-MnO_2_. We utilized a vortex machine (XH-D VORTEX) and an ultrasonic machine (XM-2200ES, Komei Ultrasonic Instruments Co., Kunshan, Jiangsu, China) to homogeneously disperse the MnO_2_ particles in 2 wt% NaA solution (1 wt% by volume) as the outer phase solution and PVA (10 wt%) as the inner phase. The inner phase solution was introduced into the system at a rate of 0.5 mL h^−1^ using a syringe pump, while the outer phase solution was introduced into the system at a rate of 1 mL h^−1^. The other preparation steps were the same as described above. This fiber was named HollowFiber-MnO_2_.

#### 2.2.3. Characterization of Hollow Fiber

The cross section of the hollow fiber was observed with an inverted optical microscope (Eclipse TS2, Nikon, Tokyo, Japan). The microscale morphology of hollow fiber was further characterized by scanning electron microscopy (SEM; S4800, Hitachi, Tokyo, Japan). The crystal structure of the fibers was analyzed by X-ray diffraction (XRD; Model: ultima IV, Riigaku, Tokyo, Japan). To study the effects of flow rates and diameters of hollow fibers, the preparation process was recorded by an inverted optical microscope (Hunan Ketianjian Photoelectric Technology Co., Ltd., AcutEye-2M-169, Changsha, Hunan, China) equipped with a CCD high-speed camera (Phantom, Wayne, NJ, USA). Image analysis of the hollow fiber was performed using ImageJ 1.54c software (http://rsb.info.nih.gov/ij/, accessed on 8 March 2023).

#### 2.2.4. Mechanical Properties of Hollow Fiber

We fixed the hollow fibers on a tensile test rig (JXLSPT-DBL, Joly Instruments Co., Ltd., Beijing, China) to test their tensile properties. The stretching speed was set to 1 mm s^−1^. The tensile strength of the fibers was tested using a universal electronic testing machine (Model 43 MTS Criterion, Eden Prairie, MN, USA). The tensile ratio was calculated according to Equation (1):

(1) $$Tensile=\frac{L-L_{0}}{L_{0}}\times 100\%$$

L_0_ is the initial length of the hollow fiber, and *L* is the breaking length of the hollow fiber. In addition, we assembled the hollow fiber into a spiderweb-like structure to test its bending property and tied the hollow fiber into a knot to test its torsional property.

In the determination of stress–strain curves of hollow fibers with different water contents, the water content (*W*%) of the hollow fibers was calculated according to Equation (2):

(2) $$W\%=\frac{m_{1}-m_{2}}{m_{1}}\times 100\%$$

m_1_ is the net weight of the fiber after losing different degrees of moisture; *m*_2_ is the dry weight of the hollow fiber. To ensure the stability of the moisture content of the hollow fibers during tensile testing, a thin protective coating of polyvinyl alcohol (PVA) was applied to the surface of the hollow fibers. The transparency of this coating ensures visibility of the fiber structure, while its barrier properties effectively slow water evaporation and ensure the moisture stability of the fiber during the test.

HollowFiber-CNTs with carbon nanotube contents of 1 wt%, 2 wt%, 3 wt%, 4 wt%, and 5 wt% were cut into 2-cm segments, respectively, and fixed on the tensile test platform to test the tensile property. The test method was the same as above.

#### 2.2.5. Measurement of Hollow Fiber Diameter

The hollow fibers were rapidly cut into fine segments using a fine cutting tool. From these segments, a representative cross-sectional sample was obtained and imaged under an optical microscope (Eclipse TS2, Nikon, Japan). To ensure the accuracy of subsequent measurements, a graduated ruler was placed next to the sample during the imaging process to provide a scale reference. Subsequently, the scale on the ruler was measured using ImageJ 1.54c software, and the exact diameter of the fibers was determined accordingly.

#### 2.2.6. Performance Testing of Wearable Fiber Sensors

We evaluated the performance of HollowFiber-CNTs using an electrochemical workstation (CHI760E, test resistor with an AC voltage of 0.1 V and an AC frequency of 1000 Hz). To improve the accuracy of the tests, the HollowFiber-CNTs were braided into a block form and encapsulated with titanium sheets and adhesive tape.

To evaluate the stability of HollowFiber-CNTs in different acid–base environments, we added hydrochloric acid solution with pH = 2, 4, and 5 and sodium hydroxide solution with pH = 8, respectively. After a certain period, the residual solution was removed using absorbent paper, and the relative resistance change (Δ*R*/R_0_) of the HollowFiber-CNTs was recorded continuously.

The samples were placed on a heating platform (Lebertek Instruments, Beijing, China, ZH35B). The temperature was gradually increased from 24 °C to 60 °C and then gradually decreased to 24 °C. During this period, continuous changes of ∆*R*/*R*_0_ were recorded to characterize the stability of the HollowFiber-CNTs over temperature changes.

We performed resistance tests on HollowFiber-CNTs with different CNT contents (1 wt%, 2 wt%, 3 wt%, 4 wt%, and 5 wt%) and calculated their conductivity (*ρ*) according to Equation (3):

(3) $$\rho =\frac{RS}{L}$$

where *R* is the resistance, *S* is the cross-sectional area of HollowFiber-CNTs, and *L* is the length of HollowFiber-CNTs.

We braided the HollowFiber-CNTs to a length of 0.5 cm, connected them to the LED strip, and recorded the luminous intensity of the bulbs. Subsequently, the HollowFiber-CNTs were stretched to observe the effect of different stretching speeds on light bulb luminescence. We chose a 3.5 cm length of HollowFiber-CNTs to be fixed in the stretching test platform, set the stretching speed to 2 mm s^−1^, recorded the changes of ∆*R*/R_0_, and obtained the sensitivity coefficient GF based on Equation (4):

(4) $$GF=\frac{∆R/R_{0}}{∆L/L_{0}}$$

HollowFiber-CNTs were attached to the finger joints, wrists, elbows, knees, and throat areas, and the data changes in ∆*R*/R_0_ were monitored using an electrochemical workstation. Meanwhile, an electromyographic (EMG) signal detection device (3IT EMGC1V1) was used to record the EMG signals. The HollowFiber-CNTs were braided into 2 cm × 2 cm squares to be taped on both sides of the biceps as electrodes and then connected to EMG electromyographic sensors using wires to collect the changes of physiological electrical signals during muscle movement. Corresponding EMG signals were converted into waveforms using backyard brain V1.4. software and exported data, then plotted using MATLAB 2018a software.

#### 2.2.7. Motion Testing of Micromotors

We investigated the motion behavior of magnetic hollow fiber micromotors using microscopy techniques. We tested the motion of magnetic hollow fiber micromotors along specific straight paths using an applied magnetic field. We sequentially preset straight paths, curved paths, complex paths, and narrow passages to observe whether the magnetic hollow fiber micromotor can move precisely and smoothly under the effect of an applied magnetic field. All tests were conducted under specific experimental conditions and parameters to ensure the reliability and reproducibility of the results.

In bubble-propelled micromotors, polydimethylsiloxane was used to make molds with complex cavities and put into the petri dish. The functionalized hollow fiber was cut into pieces and put into the cavity of a petri dish filled with H_2_O_2_ to test its motion. The reaction is based on the following chemical equation:

(5) $$2H_{2}O_{2}\overset{MnO_{2}}{\to }O_{2}+2H_{2}O$$

The concentration of H_2_O_2_ solution was varied, and the time recorded for movement over the same distance to obtain the velocity of the fiber segments at different concentrations. The data were processed using Origin 8.0 software to obtain a relationship between the concentration of the solution and the velocity of movement.

## 3. Results and Discussion

### 3.1. Fabrication and Morphology Characterization of Hollow Fibers

**Figure 1:**
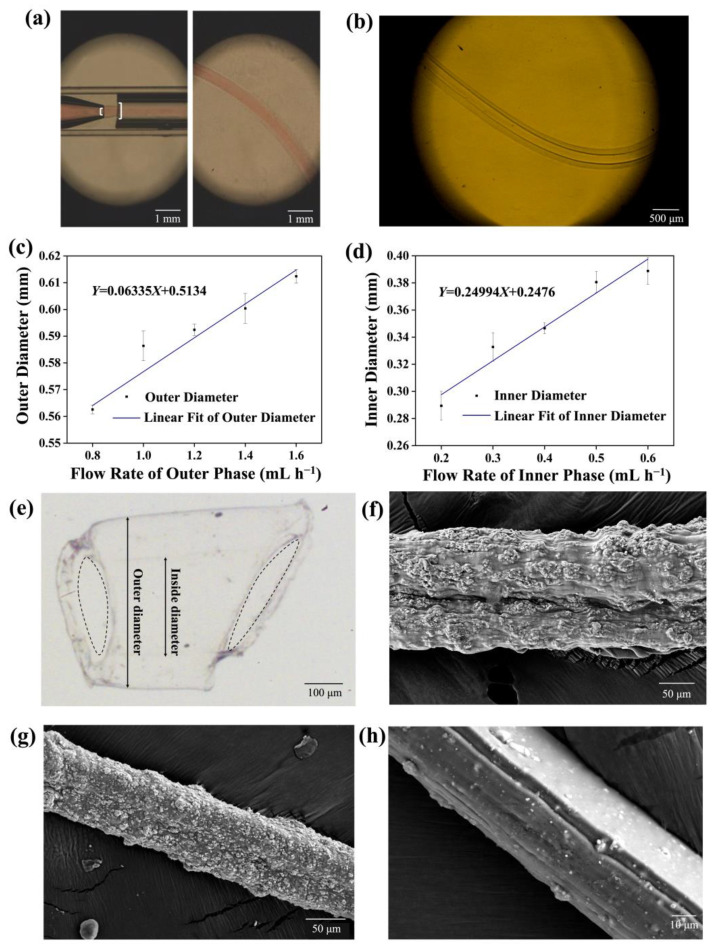
Fabrication and morphology properties of hollow fibers. (**a**) Partial enlarged detail of the internal structure of the microfluidic device and fresh-made hollow fiber (the inner diameter of left fine tip: 0.26 mm; the inner diameter of right thick tip: 0.58 mm). (**b**) Photograph of the resultant hollow fiber. (**c**) Relationship between the flow rate of the outer phase and the outer diameter of the hollow fiber. (**d**) Relationship between the flow rate of the inner phase and the inner diameter of the hollow fiber. (**e**) Cross-section of hollow fibers (hollow fiber inner diameter: ~0.269 mm; hollow fiber outer diameter: ~0.486 mm). (**f**) SEM image of a hollow fiber surface. (**g**) SEM image showing the Fe_3_O_4_ nanoparticles on the surfaces of magnetic-driven micromotors. (**h**) SEM image showing the MnO_2_ nanoparticles on the surfaces of bubble-propelled micromotors. Data are presented as mean ± SD (*n* = 3).

The hollow fibers can be fabricated according to the method shown in Scheme 1, and a clear interface (Figure 1a and Supporting Video S1) can be observed between the core solution and the shell solution. When the inner phase solution is changed to MC, HPMC, and PEG, the hollow fibers can still be prepared perfectly (Figure S1). This is because in a co-flowing microchannel, when two incompatible water phases are in contact, the growth rate of interfacial instability in the whole water jet depends on the value of interfacial tension relative to inertial force and viscous force. For this system, with an interfacial tension lower than 0.1 mN m^−1^, the capillary force between the two water phases is usually negligible, and mass transfer does not easily occur between the two phases, so two immiscible phases can be separated by a long, straight interface [42]. After solidification, we easily rinsed the inner phase with pure water and obtained the resultant hollow fiber (Figure 1b) with a perfect hollow structure.

By adjusting the flow rates of the inner and outer phase solutions, we found that the inner and outer diameters of the fibers were directly proportional to the inner phase flow rate and the outer phase flow rate, respectively (Figure 1c,d). The inner diameter of the fibers was inversely proportional to the outer phase flow rate (Figure S2a), and there was little effect between the outer diameter of the fibers and the inner phase flow rate (Figure S2b). This allowed us to precisely control the dual-phase flow rate and, thus, the thickness of the hollow fiber wall.

The electron microscope image of the hollow fiber cross-section shows the complete hollow structure, with the dotted line marking the hollow portion (Figure 1e). By observing HollowFiber-CNTs, HollowFiber-Fe_3_O_4_, and HollowFiber-MnO_2_ using scanning electron microscopy, we clearly found that carbon nanotubes, iron tetroxide nanoparticles, and manganese dioxide particles were uniformly distributed on the outer phase walls. (Figure 1f–h). Intriguingly, the surface of the resultant hollow fiber demonstrated alligator cracking. Using scanning electron microscopy (SEM) to observe the surface of HollowFiber-CNTs, we found many parallel folds on the outer wall of the hollow fiber (Figure S3a,b).

This indicates that the CNTs are arranged uniformly in the hollow fiber, so the hollow fiber appears uniformly shrunken after drying. By comparing the hollow fibers’ surfaces before and after doping with nanoparticles, we found that after doping with CNTs, the fiber walls showed almost no cracks, which might be the result of the interaction between carboxyl groups of CNTs and hydroxyl groups of calcium alginate through a hydrogen bond. However, compared to smooth surfaces, HollowFiber-CNTs show a folded structure on their surface. This wrinkled structure gives HollowFiber-CNTs a larger actual surface area. From the perspective of sensing applications, the increased surface area implies that more fiber surface regions are involved in the interaction when in contact with living organisms or other substances, thus increasing sensitivity to strain. This increased surface contact may lead to an increase in the number of conductive paths, further enhancing the sensitivity of the sensor. On a microscopic scale, this pleated structure provides additional contact points that are critical in human detection or other sensing applications, enhancing the stability and reliability of signal acquisition and thus optimizing the performance of the sensor.

We also performed a detailed crystal structure analysis of the three different fiber materials using X-ray diffraction (XRD) (Figure S4). Through fine resolution and comparison of the XRD patterns of the samples, we observed that the positions of the diffraction peaks were highly consistent with the characteristic diffraction peaks of CNTs, Fe_3_O_4_ and MnO_2_, except for the diffraction peaks specific to NaA itself. This result not only verifies the successful doping of carbon nanotubes, Fe_3_O_4_, and MnO_2_ in the calcium alginate matrix but also reconfirms that the homogeneous distribution and immobilization of these nanomaterials in calcium alginate fibers can be efficiently achieved by the microfluidic fabrication method.

### 3.2. Mechanical Properties of Hollow Fiber

By comparing the stretchability of hollow fibers prepared with different inner phase solutions (Figure S5a), we found that a hollow fiber prepared with PVA as the inner phase can be stretched from 5 cm to 11 cm, more than 220% of its original length (Figure S5b and Supporting Video S2), which is significantly higher than the others, showing its unique tensile strength. This may be because the residual PVA after cleaning was attached to the inner wall of the hollow fiber to improve the stretchability of the hollow fiber, as previously reported [43,44]. Therefore, PVA was used as the inner-phase solution in the subsequent preparation of hollow fiber.

**Figure 2:**
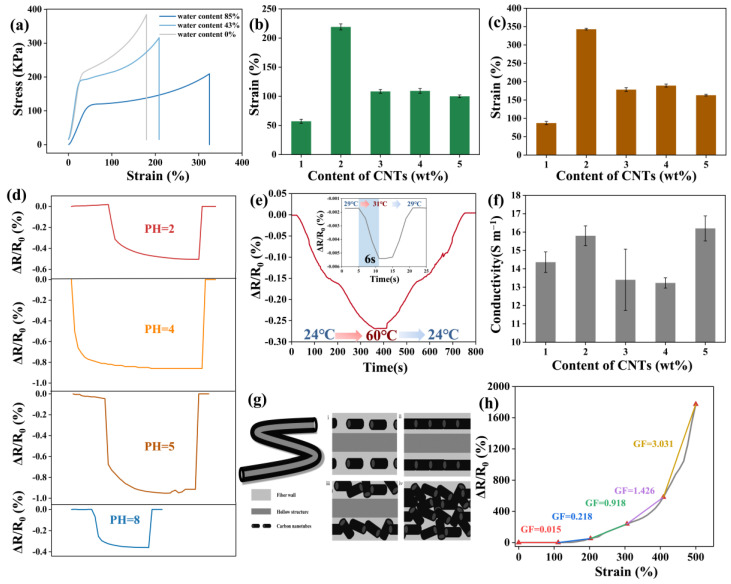
Property characterization and morphology of HollowFiber-CNTs. (**a**) Stress–strain curve of hollow fibers with different water contents. (**b**) Relationship between the content of CNTs and the electrical conductivity of hollow fiber. (**c**) Relationship between CNT content and hollow fiber tensile rate after soaking in LiCl. (**d**) Changes in the relative resistances of HollowFiber-CNTs at different pH values. (**e**) Changes in the relative resistance and response time of HollowFiber-CNTs at different temperatures. (**f**) Relationship between CNT content and hollow fiber tensile rate. (**g**) Alignment of CNTs with different contents inside hollow fiber. (**h**) Relationship between tensile rate and GF of HollowFiber-CNTs. Data are presented as mean ± SD (*n* = 3).

We also determined the stress–strain curve of hollow fiber (Figure 2a), and the results showed that the tensile strength of the hollow fiber increased, and the stretchability decreased as the water content of the fibers decreased. The tensile strength of fully dried hollow fiber can reach up to 245 kPa. The tensile strength is highly dependent on the water content of the hollow fiber. Moreover, the resultant hollow fiber can be easily assembled into a spiderweb-like network (Figure S5c) and tied together (Figure S5d), showing its good bending and torsional properties.

We then performed tensile tests on HollowFiber-CNTs with different CNT contents. When the carbon nanotube content was low, the dispersion in the fiber was good, playing the role of the enhancer. When the content reached 2%, the enhancement effect was the best. When the content continued to increase, carbon nanotubes were prone to aggregation, affecting the overall performance of the material. We found that 2% HollowFiber-CNTs exhibited the best tensile property, up to 219.05 ± 5.23% (Figure 2b). After soaking in LiCl solution, the stretchability of the HollowFiber-CNTs was significantly improved and increased to 342.9 ± 2.23% (Figure 2c).

The good mechanical characteristics of hollow fiber, together with its better wearability, provide possibilities for the subsequent development of wearable fiber sensors and self-driven water purifiers.

### 3.3. Wearable Fiber Sensor

#### 3.3.1. Stability Test of HollowFiber-CNTs

According to the experimental results, HollowFiber-CNTs also showed good stability in acid-based solutions. The ∆*R*/R_0_ recovered to its initial level after removing the acid-based solution (Figure 2d). This characteristic can also be used to monitor the solution’s pH. HollowFiber-CNTs also have good stability in a high-temperature environment. When the temperature was increased from 24 °C to 60 °C and then decreased to 24 °C the ∆*R*/R_0_ maintained a good congruent relationship with the temperature (Figure 2e). This characteristic of being able to output different ∆*R*/R_0_ signals at different temperatures can also be used for temperature monitoring. The high sensitivity, coupled with the excellent stability of HollowFiber-CNTs, is an essential property of wearable sensors when monitoring body movement.

#### 3.3.2. Electrical Conductivity of HollowFiber-CNTs

We measured the electrical conductivity of HollowFiber-CNTs with CNT contents from 1 wt% to 5 wt%, and we found that the hollow fiber with 2% and 5% CNT contents had the highest conductivities, with conductivities of 15.8 ± 0.54 S m^−1^ and 16.2 ± 0.68 S m^−1^, respectively (Figure 2f). Analyzing the possible reasons, the low content of CNTs means that they do not contact each other and do not form efficient conductive pathways (Figure 2g(i)). Therefore, they have low conductivity. With an increase in content, the direction of CNTs is consistent with the direction of fluid flow due to the action of shear force, and thus they can phase head to tail to form more effective conductive pathways (Figure 2g(ii)). With the increase in CNT content, the direction of the CNTs becomes uncontrollable and an effective conductive pathway cannot be formed (Figure 2g(iii)), causing decreased conductivity. But when the CNT content continues to increase, the CNTs will pile up in the hollow fiber; although the orientation is disordered, there are multiple conductive pathways, so the conductivity of the hollow fiber is enhanced (Figure 2g(iv)). At this point, if the CNT content is further increased, a high concentration of CNTs will augment both electrostatic interactions and van der Waals forces between the CNTs. This increase will make it more likely for CNTs to form agglomerates at a high volume rather than being dispersed within the matrix. The increase in spacing between the aggregated CNTs impedes electron hopping among them, thereby leading to a decrease in electrical conductivity.

We also found that the resistance changes of HollowFiber-CNTs maintain a relationship of positive correlation with the magnitude of their deformation rate. The luminescence intensity of LED light shows this phenomenon intuitively (Figure S6a): the higher the deformation rate, the darker the light, indicating greater fiber resistance. This is because before HollowFiber-CNTs are stretched, the CNTs are well connected and form a complete conductive pathway, so a lower resistance value can be maintained. When the hollow fiber segment is stretched, the CNTs doped in the hollow fiber are separated from each other, resulting in an elevated resistance value. By calculating the sensitivity coefficient at different tensile rates, we found that the GF value of this hollow fiber increased with increasing deformation (Figure 2h), illustrating its high sensitivity in detecting large joint movements.

#### 3.3.3. Wearable Hollow Fiber Sensors for Detecting Human Motion

The resistance of HollowFiber-CNTs changes with the deformation of the fiber. The fiber deforms when it encounters an external force, which leads to a change in the conductive pathway within the fiber, resulting in a change in the resistance value. These changes in resistance can be represented by measuring the relative change in resistance (Δ*R*/R_0_), where R_0_ is the initial resistance and Δ*R* is the change in resistance. When HollowFiber-CNTs are affixed to joints such as finger knuckles, wrists, elbows, and knees for motion detection, they deform according to the flexion angle of the joint, which leads to variations in resistance. By measuring these changes in resistance, the bending angle of the joint can be determined, thereby facilitating the detection and recognition of motion.

**Figure 3:**
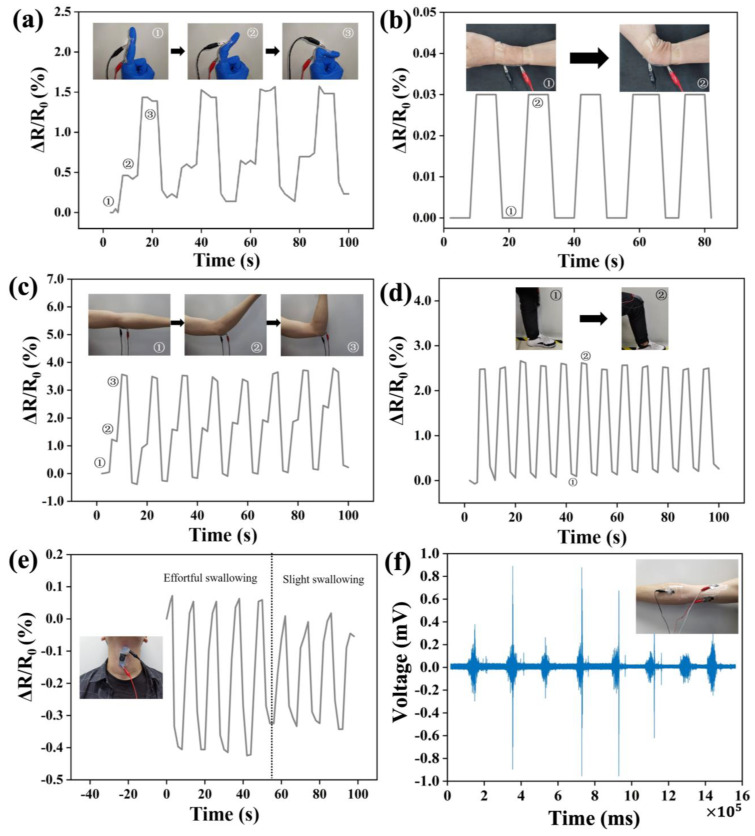
Applications of HollowFiber-CNTs for detecting human motion. (**a**) Relative resistance changes of HollowFiber-CNTs with finger joint bending to different angles. (**b**) Relative resistance changes of HollowFiber-CNTs with wrist bending to different angles. (**c**) Relative resistance changes of HollowFiber-CNTs with elbow bending to different angles. (**d**) Relative resistance changes of HollowFiber-CNTs with knee bending to different angles. (**e**) Relative resistance changes of HollowFiber-CNTs with varying degrees of swallowing. (**f**) HollowFiber-CNTs detection of electromyographic signals.

Due to the excellent stability of HollowFiber-CNTs, we demonstrated the potential practical application of HollowFiber-CNTs as wearable devices and prepared a comprehensive strain-sensing test on different joints in humans. It was found that HollowFiber-CNTs exhibited excellent sensitivity for the identification of human activity, including large body movements and weak signals. Figure 3a shows the detection performance of the HollowFiber-CNTs sensor for finger movement. When the finger was repeatedly flexed from 0° to 90° to 135°, the ΔR/R_0_ value increased correspondingly, whereas it decreased during the stabilization phase after straightening the finger. Figure 3b depicts the relationship between Δ*R*/R_0_ and bending angle as the HollowFiber-CNTs sensor monitors wrist movement. HollowFiber-CNTs sensors were also affixed to the elbow and knee joints to test their responses to large amplitude movements, showing periodic Δ*R*/R_0_ vibration curves (Figure 3c,d). The value of Δ*R*/R_0_ increased significantly with the flexion angle, so that joint movements flexing to different angles could be distinguished, showing a similar trend to finger movement.

In addition to detecting movements, strain-sensing performance was used to monitor subtle signals, including swallowing, as well as the collection of EMG signals generated in response to muscle movements. HollowFiber-CNTs were collected on the back of an adhesive film, which was affixed to the throat site. Swallowing causes an altered laryngeal position, which in turn causes the HollowFiber-CNTs to deform. We analyzed the ΔR/R_0_ signal trend across various swallowing intensities (Figure 3e). The Δ*R*/R_0_ signal change amplitude was smaller when a slight swallowing movement was made, and increased when a vigorous swallowing movement was made, which was consistent with the above results when detecting large amplitude movements. While serving as an EMG signal collection electrode, HollowFiber-CNTs showed good sensing performance and could generate obvious EMG signal waveforms (Figure 3f and Figure S6b), thus enabling the detection of muscle movement.

The above results verified the excellent sensing performance of the HollowFiber-CNTs sensor, which showed an excellent ability to identify human activities, and the profiles of these motions displayed unique morphologies and exhibited good repeatability, which guaranteed their application as wearable devices in detecting different human activities as well as physiological activities [45,46] (Table S1). To ensure that the sensors provide reliable and accurate data, they need to be used at a constant temperature to ensure environmental stability and prevent fluctuations in temperature and humidity from affecting sensor performance.

### 3.4. Micromotors

#### 3.4.1. Magnetic-Driven Micromotors

Micromotors have attracted more and more attention due to their great application value [47]. The hollow fiber-based micromotor, as an emerging direction of micromotors, is playing an important role in the small-scale machine field due to its versatile hollow structure. The resultant hollow fiber with excellent performance is qualified for use in micromotor applications.

**Figure 4:**
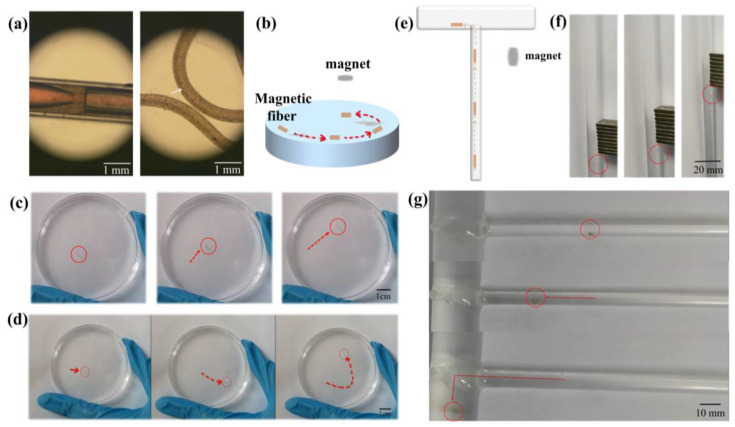
Directional movement of nano-doped hollow fiber. (**a**) Preparation process of magnetic hollow fibers and the resultant magnetic hollow fibers. (**b**) Schematic diagram of the magnetic control of directional movement of the directional micromotor. (**c**) Magnetic force controls the movement of a directional micromotor along a straight line. (**d**) Movement of the directional micromotor along a curve. (**e**) Schematic diagram showing the manipulation of the directional micromotor in a channel. (**f**) Manipulation of the directional micromotor in a vertical channel. (**g**) Manipulation of the directional micromotor in a T-shaped channel.

We prepared magnetic hollow fibers by introducing magnetic nanoparticles into the outer phase, which were uniformly distributed along the outer phase wall (Figure 4a). We then investigated the motion behavior of the magnetic hollow fiber micromotor, as shown in Figure 4b. To start with, we tested the motion of the magnetic hollow fiber micromotor along a specific linear path. The results revealed that the magnetic hollow fiber micromotor could travel to the intended destination along a predefined linear path under the effect of an applied magnetic field (Figure 4c). Similarly, the magnetic hollow fiber micromotor could move precisely and smoothly along a preset curved path (Figure 4d), demonstrating exceptional predictability, stability, and controllability. Moreover, we achieved precise manipulation of magnetic hollow fiber micromotors in complex channels (Figure 4e). As shown in Figure 4f, the magnetic hollow fiber micromotor was able to move vertically in a narrow channel under magnetic manipulation. Additionally, the magnetic hollow fiber micromotors could successfully achieve precise and directional motion in intricate T-shaped channels using external magnetic fields (Figure 4g and Supporting Video S3). In summary, magnetic hollow fiber micromotors offer robust maneuverability and directional mobility, thus holding great promise for various applications, such as drug delivery, targeted therapy, and small machines.

#### 3.4.2. Bubble-Propelled Micromotors

Hydrogen peroxide (H_2_O_2_) has been widely used in several viable applications at low concentrations, such as a sensing platform for analyzing targets [48]. H_2_O_2_ is considered a powerful oxidizing agent. Calcium alginate (CaAlg) is a salt obtained by reacting alginate with calcium ions. Notably, the calcium ions in calcium alginate exhibit stability to H_2_O_2_ and do not participate in redox reactions. Therefore, it can be inferred that the chemical state of calcium alginate is relatively stable in H_2_O_2_ solution. The configuration of microtubules plays a pivotal role in enabling the generation, accumulation, and release of bubbles, which is essential for the efficacious propulsion of micromotors [49,50].

Therefore, we embedded manganese dioxide (MnO_2_) particles into the walls of the hollow fibers to prepare functionalized fibers with the function of catalyzing H_2_O_2_ decomposition. Due to the capillary mechanism, H_2_O_2_ will be adsorbed by the interior of the hollow fiber and decomposed into oxygen by the catalytic effect of MnO_2_. This newly generated oxygen rapidly accumulates within the limited space of the hydrogel, leading to an increase in internal pressure and creating a localized pressure gradient. Due to this pressure gradient, the hydrogel fibers are driven toward areas of lower pressure when oxygen is generated inside the fibers. The external MnO_2_ also catalyzes the decomposition of H_2_O_2_ to produce oxygen bubbles; however, surface tension causes these bubbles to adhere tightly to the outer wall of the fiber. Since these bubbles do not form in a confined space where directional thrusts can be generated, they have less effect on the dynamics of the fibers (Figure S7a).

**Figure 5:**
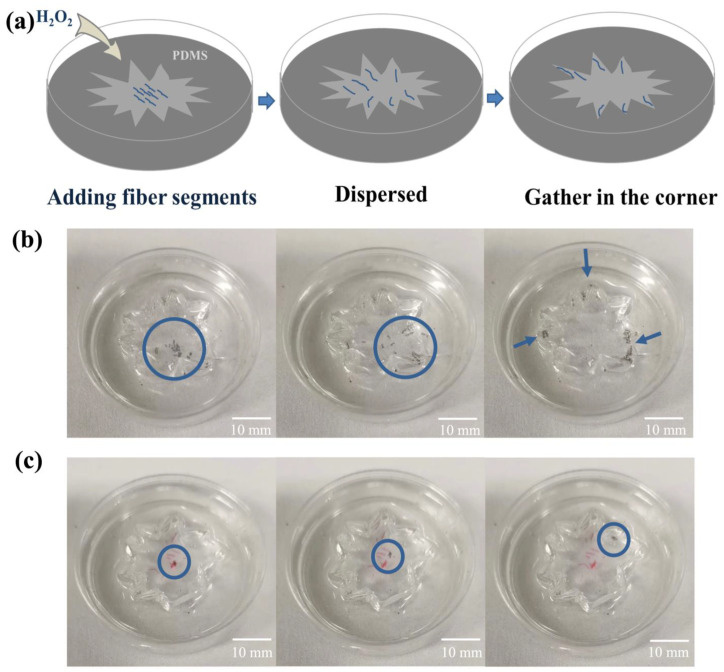
The bubble-driven hollow fiber micromotor. (**a**) Schematic diagram of the bubble-driven hollow fiber micromotor. (**b**) Movement of hollow fibers with MnO_2_ on the inner wall in H_2_O_2_. (**c**) Movement of hollow fibers with MnO_2_ on the inner wall and general hollow fibers in H_2_O_2_.

Therefore, the oxygen bubbles can push the hollow fiber forward, showing a good performance of the hollow fiber-based micromotor. To test the ability of bubble-driven micromotors to enter small and deep locations, we placed them inside a mold filled with hydrogen peroxide solution (Figure 5a), and the results indicated that the bubble-driven micromotor immediately spread out toward the corner and finally gathered deep in the corner. (Figure 5b and Supporting Video S4). To rule out coincidental factors, we synthesized a batch of undoped MnO_2_ hollow fiber samples as a blank control (the red part in Figure 5c), which were mixed with the experimental group and put into the molds according to the experimental procedure described before. The results clearly showed that the locations of the hollow fiber segments without MnO_2_ did not change, whereas the bubble-driven micromotor moved toward the periphery and finally stayed deep in the corners (Figure 5c and Supporting Video S5). By changing the concentration of the hydrogen peroxide solution, we found that the speed of the bubble-driven micromotor movement was accelerated with an increase in hydrogen peroxide concentration (Figure S7b). This is because the higher concentration of H_2_O_2_ can generate bubbles more quickly after it is decomposed, enabling them to push the micromotors more effectively. The movement of this bubble-driven micromotor can deliver efficient and robust performance [51,52]. The micromotor can move in 5 wt% H_2_O_2_ solution at a speed of 615 μm s^−1^, which is higher than that of some other micromotors of the same type that have been reported so far (Figure S7c).

## 4. Conclusions

In this work, we describe a microfluidic method based on coaxial glass capillaries for fabricating nanomaterial-doped functional hollow fibers. This method features a simple, smooth, stable, continuous, and precisely controlled process that avoids system clogging. Utilizing the characteristics of microfluidic laminar flow as well as the shear forces acting in the microchannels, we achieve uniform dispersion of nanoparticles to enable hollow fiber with favorable mechanical, electrical, and magnetic operabilities. By doping CNTs in the hydrogel hollow fibers, we successfully prepare wearable sensors exhibiting superior electrical conductivity (15.8 S m^−1^), strong flexibility (342.9%), versatility, and high sensitivity at high deformation rates. This CNT-doped hollow fiber-based wearable sensor can be used to monitor human motions and collect physiological electrical signals. As well as wrist movements, elbow and knee movements could also be detected, and minor movements, such as swallowing and finger joint movements, could also be successfully monitored. Moreover, by doping Fe_3_O_4_ nanoparticles into hydrogel fibers, we fabricated magnetic-driven micromotors capable of directional motion along the trajectory of a magnet. Owing to their small size, these micromotors could navigate through narrow channels. In addition, manganese dioxide nanoparticles were embedded into the hollow fiber walls to create bubble-propelled micromotors. When put into a hydrogen peroxide environment, the micromotors with MnO_2_-doped fiber walls could reach high-speed self-propulsion (615 μm s^−1^) and were capable of penetrating tiny and hard-to-reach corners. This technology provides a highly flexible and controllable preparation method, which makes it possible to precisely dope different functional materials, and not only realizes the multifunctionality of hollow fibers, but also the easy weaving and integration of these hollow fibers enables them to be used in the preparation of smart fabrics with multiple functions. This innovative technology provides a powerful tool for designing and preparing novel materials with customized functions. It sheds light on the design and development of wearable electronic devices, soft robots, micromachines, and future applications in biomedical engineering.

## Data Availability

Data are available upon reasonable request.

## Supplementary Materials

The following supporting information can be downloaded at: https://www.mdpi.com/article/10.3390/bios14040186/s1, Figure S1: Fabrication and morphology of hollow fibers; Figure S2: Flow velocity versus hollow fiber diameter; Figure S3: Localized enlarged image of HollowFiber-CNT surface morphology taken by scanning electron microscope; Figure S4: XRD images of hollow fibers; Figure S5: Mechanical properties of hollow fiber; Figure S6: Electrical properties of hollow fibers; Figure S7: Bubble-propelled micromotors; Table S1: Comparison of electrical conductivity of hydrogel fibers of different networks with human detection function; Video S1: Preparation process of hollow fiber; Video S2: Stretching process of hollow fiber; Video S3: Manipulation of directional micromotor in T-shaped channel; Video S4: Movement of hollow fibers with MnO_2_ on the inner wall in H_2_O_2_; Video S5: Movement velocity of bubble-driven hollow fiber micromotor in H_2_O_2_ solution with different concentration.
