# Supplementary material for: Multi-Functional Nano-Doped Hollow Fiber from Microfluidics for Sensors and Micromotors
Source: Biosensors (Basel). 2024 Apr 10;14(4):186. doi: 10.3390/bios14040186 (PMC11048216; doi:10.3390/bios14040186)
Supplement: Supplementary file 1 [file biosensors-14-00186-s001.zip › SI.pdf]

Multi-functional Nano-doped Hollow Fiber from Microfluidics for Sensors and Micromotors

Yanpeng Wang[1], Zhaoyang Wang[1], Haotian Sun[1], Tong Lyu[1], Xing Ma[2], Jinhong Guo[3*], Ye Tian[1,4*]

[1] College of Medicine and Biological Information Engineering, Northeastern University, 110169 Shenyang, China.

[2] School of Materials Science and Engineering, Harbin Institute of Technology (Shenzhen), Guangdong, Shenzhen, 518055, China.

[3] School of Sensing Science and Engineering, Shanghai Jiao Tong University, Shanghai 200240, China.

[4] Foshan Graduate School of Innovation, Northeastern University, 528300, Foshan, China.

*Corresponding author: guojinhong@sjtu.edu.cn, tianye@bmie.neu.edu.cn

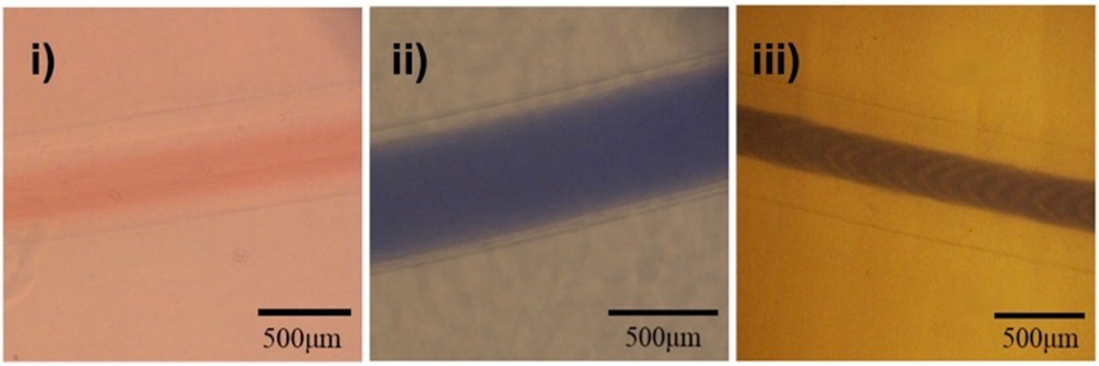

Fig S1. Fabrication and morphology of hollow fibers. Different systems for the fabrication of hollow fiber. The inner phase solutions are MC (i), HPMC ii) and PEG (iii), respectively.

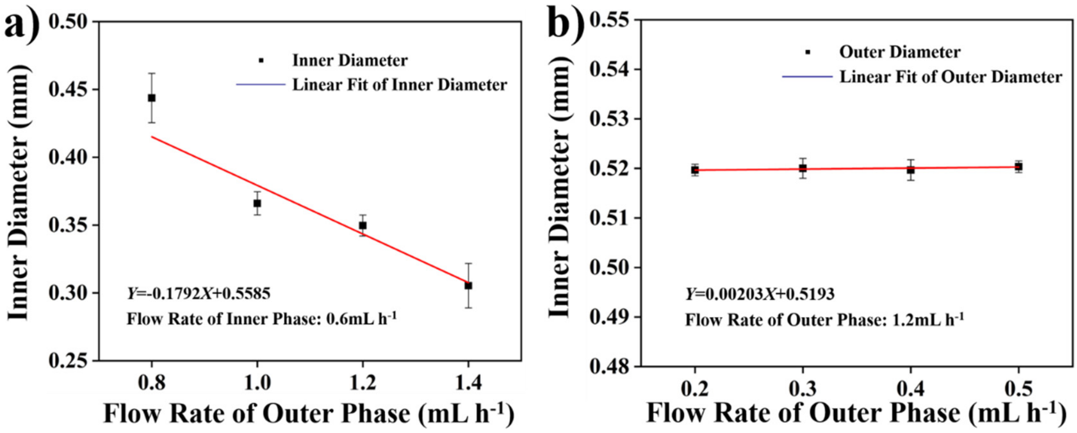

Fig S2. Flow velocity versus hollow fiber diameter. a) Relationship between the outer phase flow rate and the inner diameter of the hollow fiber. b) Relationship between the inner phase flow rate and the outer diameter of the hollow fiber.

a) b)

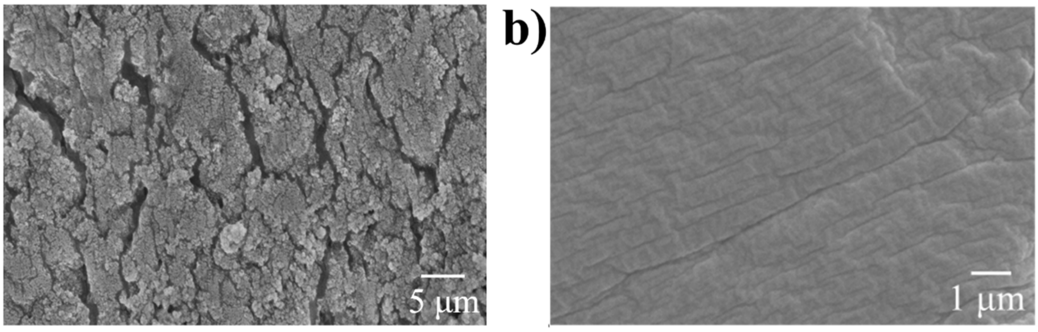

Fig S3. Localized enlarged image of HF-CNT surface morphology taken by scanning electron microscope. (a) SEM image of localized surface of HF-CNT magnified 4800 times. (b) SEM image of localized surface of HF-CNT magnified 24000 times.

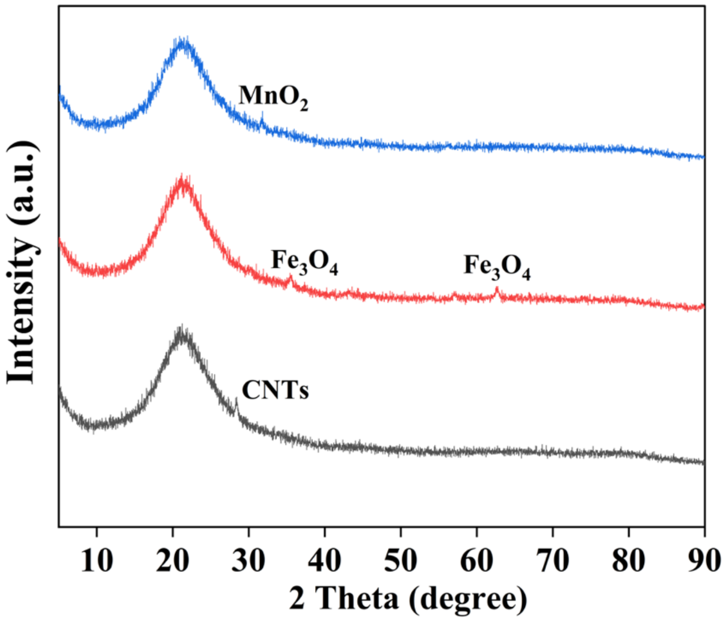

Fig S4 XRD images of hollow fibers.

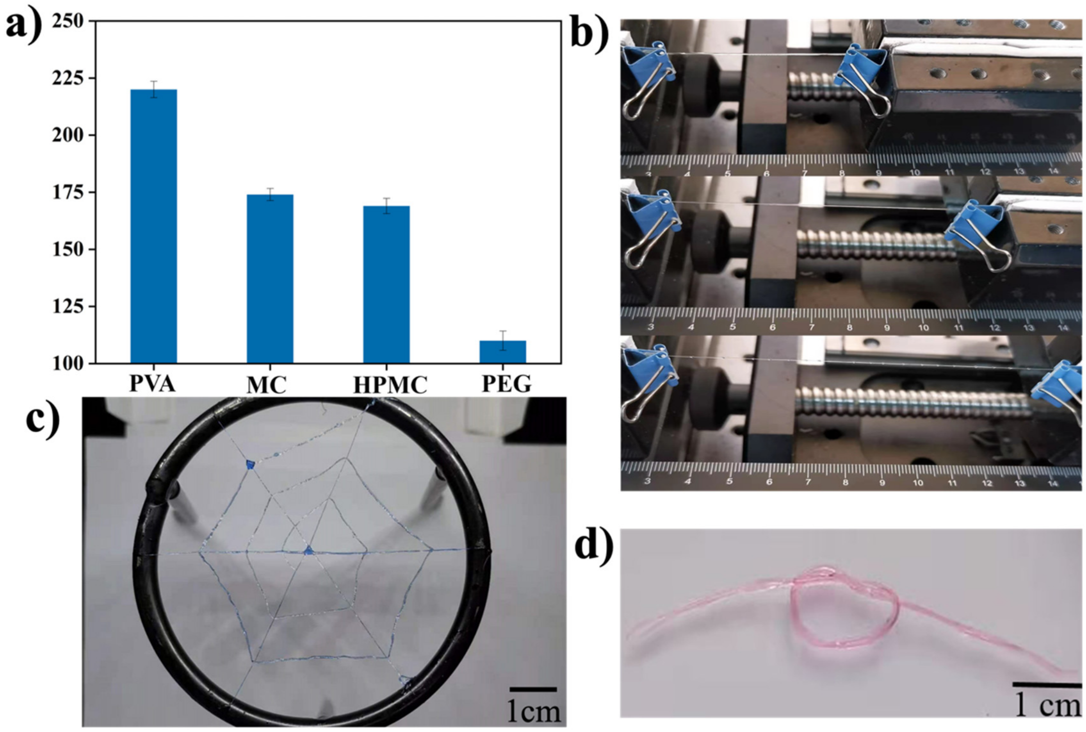

Fig. S5 Mechanical properties of hollow fiber. a) Tensile properties of hollow fibers prepared with different internal phase solutions. b) Tensile test of hollow fiber. c) Bending properties of hollow fiber. d) Torsion properties of hollow fiber.

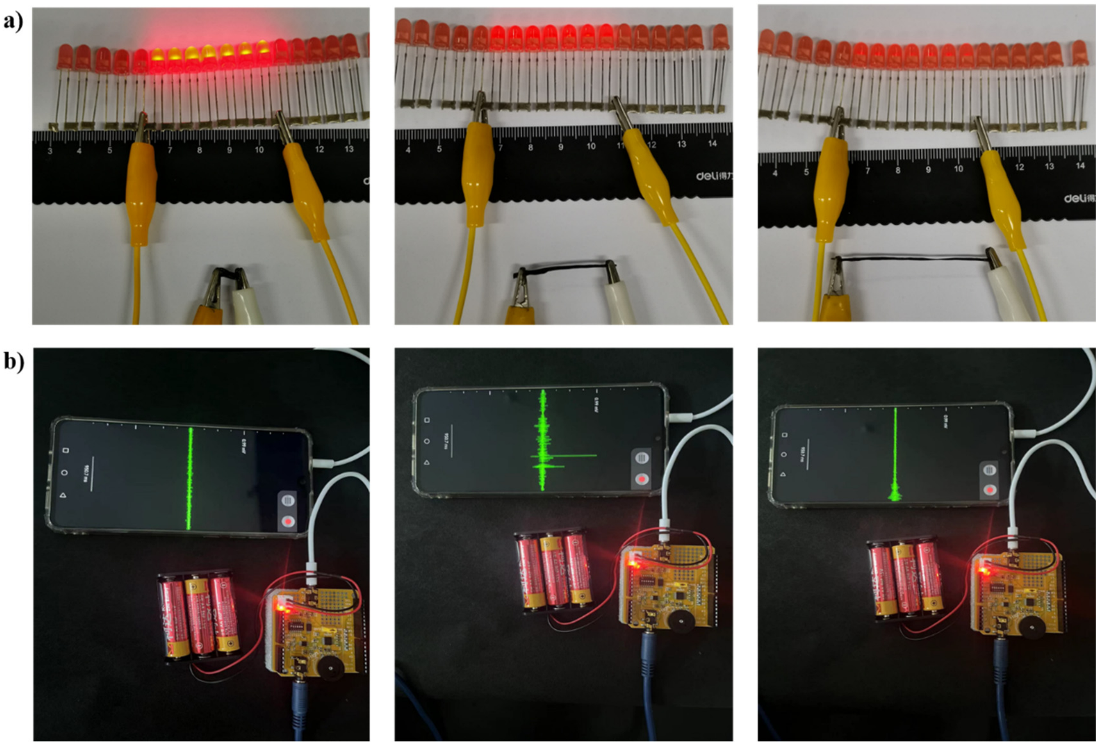

Fig. S6 Electrical properties of hollow fibers. a) The ability of hollow fiber to light up LED lamps under different degrees of stretching. b) Hollow fiber for monitoring of EMG signals.

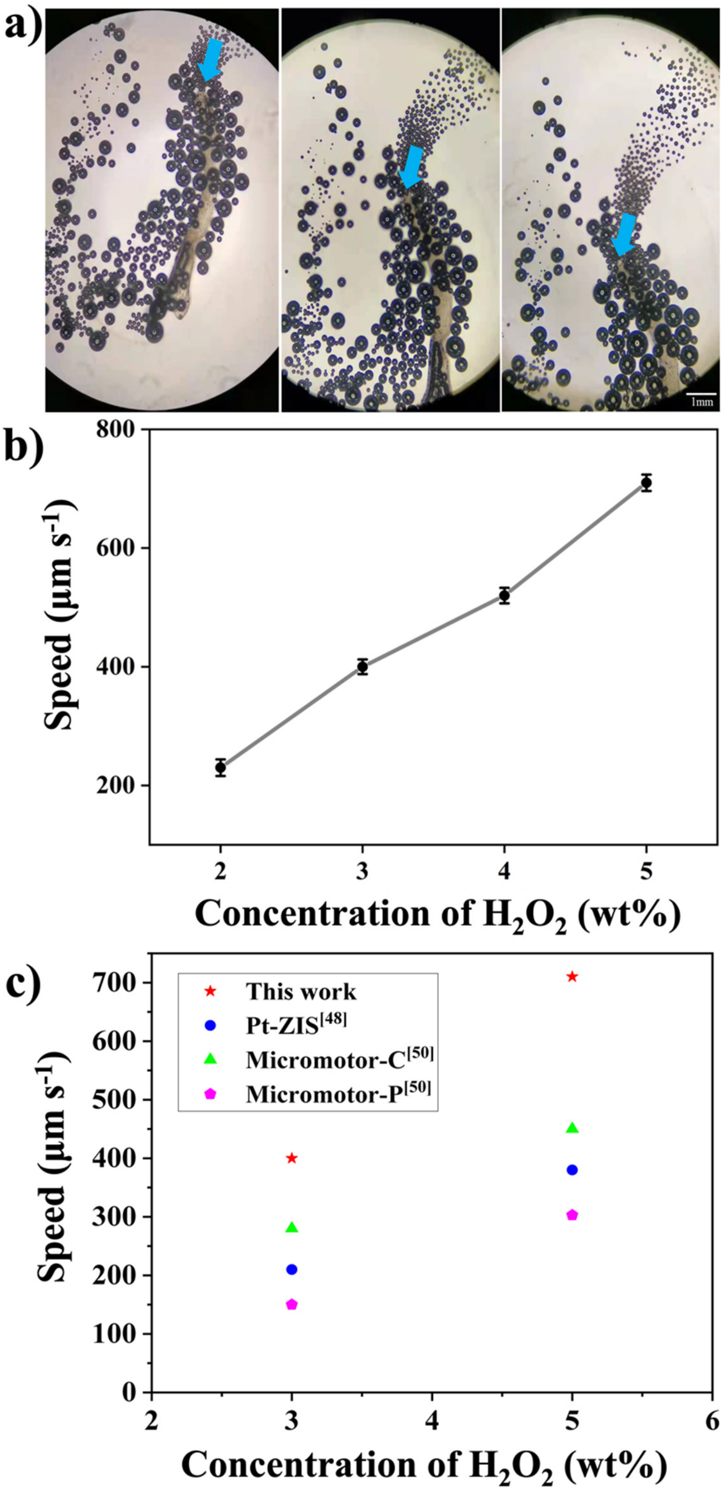

Fig S7 Bubble-propelled micromotors. a) Movement of hollow fiber micromotor with MnO_2. b) Movement velocity of bubble-driven hollow fiber micromotor in H_2O_2 solution with different concentration. c) Comparison of movement velocity of hydrogel fiber micromotors with different networks in different concentrations of H_2O_2 solution. Data is presented as Mean±SD (n=3).

Hydrogel network	Method	Conductivity (S m^{-1})	Monitoring of motion signals	Monitoring of physiological signals	Ref
AAM/NAGA	Continuous dry-wet spinning	0.69	YES	-	[45]
AAM/APhe	One-step coaxial wet-spinning	10.59	YES	-	[46]
HF-CNTs	microfluidic	15.8	YES	YES	The work

Table S1. Comparison of electrical conductivity of hydrogel fibers of different networks with human detection function.

Supporting Videos

S1 Preparation process of hollow fiber.

S2 Stretching process of hollow fiber.

S3 Manipulation of directional micromotor in T-shaped channel.

S4 Movement of hollow fibers with MnO_2 on the inner wall in H_2O_2.

S5 Movement velocity of bubble-driven hollow fiber micromotor in H_2O_2 solution with different concentration.
